# Combined Ruxolitinib and Venetoclax Treatment in a Patient with a *BCR-JAK2* Rearranged Myeloid Neoplasm

**DOI:** 10.1155/2021/2348977

**Published:** 2021-07-28

**Authors:** Coen J. Lap, Samah Nassereddine, Min-Ling Liu, Victor E. Nava, Anita Aggarwal

**Affiliations:** ^1^The George Washington University Medical Faculty Associates, Washington, DC, USA; ^2^Washington DC Veterans Affairs Medical Center, Washington, DC, USA

## Abstract

Hematological malignancies with a *BCR-JAK2* rearrangement have been described only sporadically in the literature over the last three decades. Although most patients suffer from a chronic myeloid neoplasm with marked eosinophilia, the clinical presentation varies significantly and can even manifest as a lymphoid malignancy. In this case report, we present a patient with a therapy-related *BCR-JAK2*^*+*^ myeloid neoplasm with extensive extramedullary disease localizing in the lymph nodes. While treatment with a JAK2 inhibitor (ruxolitinib) was not able to stop disease progression, combination treatment with inhibitors of both JAK2 and BCL2 (venetoclax) resulted in disease control for over 1.5 years. Combining these two inhibitors might be strategic in these patients, not only because BCL2 is a downstream target of JAK/STAT signaling but also because BCL2 is crucial for JAK2 inhibitor resistance. The recent inclusion of *JAK2*-rearranged malignancies in major classification systems and guidelines emphasizes the importance of not only getting a better understanding of the clinical phenotype of these rare disorders but also of identifying alternative treatment options for patients ineligible for allogeneic stem cell transplantation. Considering the low toxicity of combination treatment with these two small molecule inhibitors, this regimen could be further explored in future studies.

## 1. Introduction

Activating mutations in Janus kinase 2 *(JAK2)*, which include *JAK2* V617F and mutations in exon 12, are found in a high proportion of patients diagnosed with chronic myeloproliferative neoplasms (MPN), including >95% of patients with polycythemia vera and approximately 55–65% of patients with essential thrombocytosis and primary myelofibrosis [1]. While these mutations are considered crucial molecular events in the pathogenesis of these disorders, rare chromosomal translocations involving *JAK2* have been described in patients suffering from both myeloid and lymphoid malignancies [2, 3]. The t(9; 22) (p24.1; q11.2) is a recurrent rearrangement that juxtaposes the *Breakpoint Cluster Region* gene (*BCR*; 22q11.2) to the *JAK2* gene (9p24.1), which leads to the formation of a *BCR-JAK2* fusion [4]. While this translocation has been identified in patients diagnosed with acute myeloid leukemia (AML) and B-cell acute lymphoblastic leukemia (B-ALL), most case reports have described it as the sole genetic abnormality observed in patients suffering from a clinical disease that resembles myeloid neoplasms, including MPN, myelodysplastic syndrome (MDS), or MPN/MDS overlap syndromes and is often associated with significant extramedullary hematopoiesis and/or bone marrow failure [2, 3, 5–8]. In most patients, the disease is highly therapy-resistant with rapid progression occurring within the first couple of months after diagnosis [9]. While JAK2 inhibitors have been suggested to be of some benefit in controlling disease, so far, long-term remissions have only been achieved after allogeneic stem cell transplantation (allo-SCT) [10, 11]. Here, we describe a case of a patient suffering from a therapy-related *BCR-JAK2*^*+*^ myeloid neoplasm with significant disease localized in his lymph nodes. While monotherapy with a JAK2 inhibitor (ruxolitinib) was not able to control disease, combined treatment with inhibitors of JAK2 and BCL2 (venetoclax) resulted in disease control for over 1.5 years. A rationale for combining these two small molecule inhibitors is provided as an additional option, which could be further explored for patients with this rare hematological malignancy, especially when allo-SCT is not feasible.

## 2. Case Presentation

**Figure 1:**
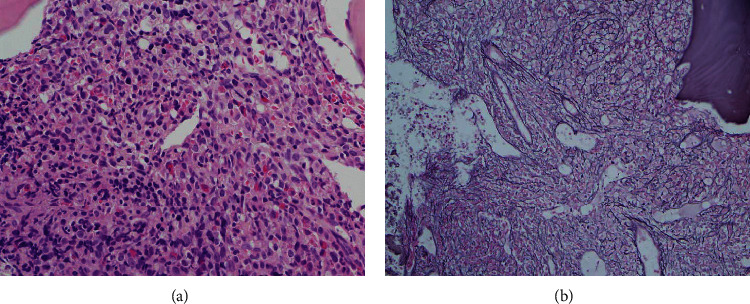
Bone marrow biopsy showing a hypercellular marrow with marked myeloid hyperplasia with left shift ((a) hematoxylin and eosin stain, 400x) and grade 3 fibrosis ((b) reticulin stain, 200x).

**Figure 2:**
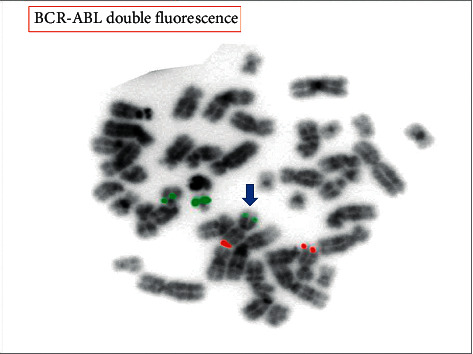
Fluorescent in situ hybridization with probes for *BCR* (22q11.2, green signal) and *ABL1* (9q34.1, red signal) was negative for a *BCR-ABL1* rearrangement. However, a third signal for the *BCR* probe (green signal, arrow) was observed, suggesting partial or complete gain of chromosome 22 or a translocation that involves *BCR* with a partner gene other than *ABL1*. Additional testing with a probe for *JAK2* (9p24.1, not shown) suggested the presence of a *BCR-JAK2* fusion, which was confirmed with RNA sequencing.

**Figure 3:**
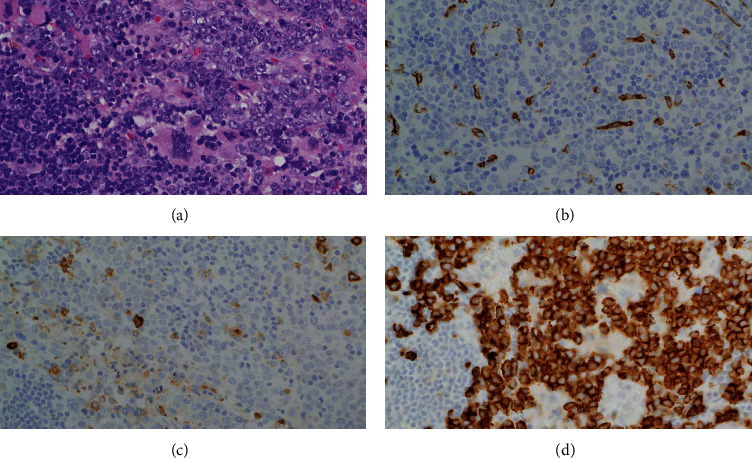
Lymph node showing extensive extramedullary involvement by a myeloproliferative neoplasm with numerous megakaryocytes ((a) hematoxylin and eosin stain, 400x), no increase in blasts ((b) CD34 immunostain, 400x), or significant myeloid cells ((c) MPO immunostain, 400x) but with prominent erythroid precursors ((d) CD71 immunostain, 400x).

A 54-year-old male presented initially in February 2013 with left axillary lymphadenopathy. He was diagnosed with stage IIA nodular lymphocyte predominant Hodgkin lymphoma (NLPHL) and received two cycles of ABVD (adriamycin, bleomycin, vinblastine, and dacarbazine) before he became lost to follow-up. In March 2014, he presented again with biopsy-proven relapsing NLPHL. A bone marrow aspirate was performed at that time, revealing an unremarkable flow analysis and a normal karyotype (46XY [12]). He received three cycles of R-EPOCH (rituximab, etoposide, prednisone, vincristine, cyclophosphamide, and doxorubicin) with additional radiation therapy to the left axillary region for residual disease. He remained in complete remission until May 2018, when he presented with fatigue, a 25 kg weight loss over three months and was found to be anemic with a hemoglobin (Hgb) of 7.2 g/dL, platelet count (Plt) of 159 × 10^3^/*µ*L, and a white blood cell count (WBC) of 6.1 × 10^3/^*µ*L. Peripheral blood smear revealed a neutrophilic left-shift with 3% myelocytes, 2% metamyelocytes, and 6% eosinophils. Evaluation of the red blood cells (RBC) revealed marked anisopoikilocytosis, teardrop cells, and 2% nucleated RBC. No circulating blasts were seen. FDG-PET/CT displayed extensive FDG-avid lymphadenopathy (SUV_max_∼10) with splenomegaly measuring 18.4 cm. Bone marrow (BM) biopsy demonstrated a hypercellular BM with left-shifted myeloid hyperplasia but less than 1% blasts (Figure 1(a)), an increased myeloid to erythroid ratio (>5 : 1) and diffuse grade 3 reticulin fibrosis (Figure 1(b)). Cytogenetics revealed a 46XY, del(6) (p21.2p24), t(22; 9; 11) (q11.2, p24, p11.2) [11]/46XY, del(9) [9] karyotype with fluorescent in situ hybridization (FISH) analysis showing absence of a *BCR-ABL1* rearrangement, but three copies of *BCR* (Figure 2). The third copy of *BCR* was identified to be on the short arm terminus of chromosome 9 resulting in a *BCR-JAK2* fusion as suggested with an additional *JAK2* (9p24.1) probe (not shown). The rearrangement was confirmed with RNA sequencing, which revealed a break in exon 1 of *BCR* (nucleotide 1107 and codon 369) and a break in exon 17 of *JAK2* (nucleotide 2171 and codon 724) leading to an in-frame fusion. The resulting product included the coiled-coil oligodimerization domain of *BCR* as well as the area distal to the pseudokinase domain (JH2) of *JAK2*, thereby maintaining the active tyrosine kinase domain (JH1) in exon 19. Additional mutation analysis was negative for *JAK2* V617 F or alterations in *CALR*, *PDGFRA*, *PDGFRB*, or *FGFR1* but did reveal an alteration in *CD36* (c.157_158AA > *G_*p.N53 fs ^*∗*^ 24). Excisional biopsy of a lymph node ruled out lymphoma, but revealed extensive extramedullary involvement of the same myeloid malignancy with prominent erythroid precursors, atypical megakaryocytes, and left-shifted myeloid cells without increase of blasts (Figure 3). Importantly, the same *BCR-JAK2* translocation was identified in the biopsy and confirmed with RNA sequencing.

Pending the results of the molecular analysis, the patient was started on the *BCR-ABL1* tyrosine kinase inhibitor (TKI) imatinib mesylate 400 mg once daily due to an initial concern of CML. Because of limited response and upon identification of the *BCR-JAK2* translocation, treatment was switched to ruxolitinib and uptitrated to 20 mg twice daily. An early referral for allo-SCT was discussed, but the patient decided to postpone this. While initially an improvement in blood counts was observed (Hgb, 10.8 g/dL; Plt, 180 × 10^3^/*µ*L; WBC, 8.1 × 10^3/^*µ*L) with a reduced need for transfusions, he developed worsening anemia (Hgb, 7.8 g/dL), thrombocytopenia (Plt, 30 × 10^3^/*µ*L), and leukocytosis (WBC, 17.6 × 10^3/^*µ*L) with an elevated LDH (680 U/L) in January 2019. A peripheral smear revealed 6% promyelocytes, 3% metamyelocytes, 6% eosinophils, and 2% blasts, but repeated bone marrow evaluation only showed diffuse fibrosis and the previously identified *BCR-JAK2* translocation. The spleen remained enlarged at 19 cm on imaging. Ruxolitinib was continued, and because of disease progression, venetoclax was initiated and uptitrated to 400 mg daily. At that point, he was evaluated for allo-SCT but was found ineligible due to diminished pulmonary function. After several weeks, blood counts improved, and the patient became transfusion-independent and EPO support was discontinued. Over time, his blood counts normalized (Hgb, 12.4 g/dl; Plt, 167 × 10^3^/*µ*L; WBC, 5.7 × 10^3^/*µ*L with 2% eosinophils) with repeat imaging, six months after the start of venetoclax, revealing a significant decrease in lymphadenopathy and a reduction in the spleen size to 13.1 cm. A new BM biopsy showed stable disease with extensive fibrosis, no increase in blasts, but persistence of the *BCR-JAK2* fusion and the *CD36* alteration, as well as a new *TET2* mutation (c.3782 G > *A*_p.R1261H). During this time, the patient tolerated the combination regimen well without development of infections, need for blood product support, or hospitalizations. Although no cytogenetic response was observed, the treatment significantly improved his quality of life. He remained stable under this combination treatment until May 2020, at which point he presented with abdominal complaints and renal failure as a result of bulky lymphadenopathy and splenomegaly due to disease progression. Ruxolitinib and venetoclax were discontinued. Blood counts revealed Hgb, 11 g/dL; Plt, 170 × 10^3^/*µ*L; and WBC, 12.1 × 10^3^/*µ*L with a peripheral smear showing left-shifted hematopoiesis with 4% eosinophils and 1% blasts. Renewed bone marrow analysis only revealed fibrosis without a significant increase in blasts. The patient was started on azacytidine and re-evaluated for allo-SCT. However, allo-SCT was further postponed as a result of the patient contracting COVID-19. During that period, azacytidine was continued, and ruxolitinib was restarted. Unfortunately, the disease progressed with worsening bone marrow failure, without signs of transformation, and he passed away several months later.

## 3. Discussion

The 2017 revision of the World Health Organization (WHO) Classification of Myeloid Neoplasms and Acute Leukemia included a new provisional entity “*Myeloid Neoplasm with PCM1-JAK2 Rearrangement*” in the category of “*Myeloid/Lymphoid Neoplasms Associated with Eosinophilia and Rearrangement of PDGFRA, PDGFRB, or FGFR1, or with PCM1-JAK2*” [12]. While the patient described here does not have a hematological malignancy with a *PCM1-JAK2* rearrangement, many experts in the field advocate to classify those rare cases with a *JAK2* rearrangement, including gene partners other than *PCM1*, in the same category because of similar clinical features [13]. This is also highlighted by the inclusion of patients with a *BCR-JAK2* rearrangement in the latest version of the U.S. National Comprehensive Cancer Network (NCCN) guidelines for “Myeloid/Lymphoid Neoplasms with Eosinophilia and Tyrosine Kinase Fusion Genes” (version 3.2021). Regardless of the classification, the presence of a *BCR-JAK2* translocation has not been linked to a specific clinical phenotype although most patients present as a chronic myeloid neoplasm with marked eosinophilia (∼50–70%) (Table 1). However, eosinophilia can be notably absent and is not a requirement for the diagnosis. Additionally, *BCR-JAK2* rearrangements are sometimes identified in the new provisional entity of Philadelphia chromosome-like ALL, which are characterized by activated tyrosine kinase signaling as well as JAK/STAT signaling but lack the presence of a *BCR-ABL1* rearrangement [19]. The clinical heterogeneity may be the result of both disease factors (additional mutations and subclones) as well as existing host factors (germline allele diversity). Our patient presented with symptoms of a myeloid malignancy with a newly acquired *BCR-JAK2* rearrangement, four years after treatment for relapsing NLPHL. Considering that the patient had a normal bone marrow evaluation at the time of his relapsing NLHPL and was subsequently exposed to cytotoxic chemotherapy, including etoposide and cyclophosphamide, it supports a diagnosis of a therapy-related malignancy. It is likely that clonal evolution and expansion over time have resulted in a secondary myeloid neoplasm. Although the clinical significance of a *CD36* N53 fs^*∗*^24 mutation is not clear at the moment, it is important to mention that it has been identified in patients with mixed phenotype acute leukemia's (MPAL) [20]. More importantly, the R1261H missense mutation in the epigenetic modifier *TET2* is a recurrent abnormality identified in (therapy-related) myeloid malignancies and is considered a driver of clonal hematopoiesis [21].

As a result of the t(9; 22) (p24.1; q11.2), a fusion gene is formed in which the oligodimerization domain of *BCR* is juxtaposed to the *JAK2* tyrosine kinase domain resulting in constitutive activation of the JAK/STAT signaling pathway, which promotes cellular proliferation, differentiation, and growth [4, 22]. JAK inhibitors such as ruxolitinib (JAK1/2) have been used successfully in patients with *JAK2*-mutated MPN and, as such, have been proposed as a possible treatment for patients with a *BCR-JAK2*^*+*^ myeloid malignancy [10, 11]. Unfortunately, responses seen in the handful of patients treated to date have been mixed with some progressing rapidly and others obtaining a complete cytogenetic response. However, even cytogenetic responses are usually short-lived due to the development of *JAK2* inhibitor resistance. Although our patient had an initial response after the start of ruxolitinib, he progressed five months later, prompting initiation of the BCL2 inhibitor venetoclax. Combining these two inhibitors may be strategic since downstream transcriptional targets of JAK/STAT signaling include antiapoptotic members of the BCL2 family, such as BCL2 and BCL-xl [23–25]. Several preclinical studies have suggested a beneficial synergistic effect of combining these two inhibitors in patients with *JAK2*-mutated hematological malignancies [25–27]. Moreover, resistance to JAK2 inhibitors has been attributed to overexpression of both BCL2 and BCL-xl. Although our patient did not develop a cytogenetic remission, the combination treatment stabilized the disease for over 1.5 years, achieving normal blood counts and significant reduction in splenomegaly and lymphadenopathy before final progression.

Even with combination treatment, our patient eventually developed progressive disease and succumbed before he was able to undergo allo-SCT. Although combined treatment might be able to control disease for a significant period of time, even when monotherapy with a JAK2 inhibitor fails, only allo-SCT results in long-term disease control, affirming the need for early referral. It needs to be emphasized that TKIs used for the treatment of *BCR-ABL1*^*+*^ CML have not shown to be of any benefit and should be avoided in the treatment of these patients [11]. Considering the rarity of hematological malignancies with a *BCR-JAK2* translocation, it remains important to report cases to disseminate clinical experience, which may allow better characterization of the clinical phenotype and provide beneficial insights regarding targeted therapy regimens for patients who are not eligible for allo-SCT.

## 4. Conclusion

*BCR-JAK2*-rearranged hematological malignancies are rare disorders that can have a variable clinical presentation but should be classified as a “Myeloid/Lymphoid Neoplasms with Eosinophilia and Gene Rearrangement.” Because eosinophilia can be discrete, or even absent, it highlights the importance of cytogenetic analysis in all patients that are presenting with a myeloid neoplasm. Combination treatment with inhibitors of JAK2 and BCL2 has minimal toxicity and can be beneficial in controlling disease.

## Figures and Tables

**Table 1:** Summary of the literature regarding described phenotype, treatment, and response duration of patients with a *BCR-JAK2* fusion.

Reference	Age	Sex	Disease	Treatment	Follow-up
Griesinger et al. [2]	63	F	CML-like MPD, myeloid blast crisis	Imatinib, hydroxyurea, interferon-alpha	Complete hematological response. 20 months after diagnosis, she developed myeloid blast crisis and died.
Kantarcioglu et al. [3]	64	F	MDS	Not described	Died 3 months after diagnosis
Cuesta-Domínguez et al. [4]	58	M	B-ALL	Not described	Not described
Cirmena et al. [5]	67	F	AML	Not described	Not described
Tirado et al. [6]	14	M	B-ALL	Not described	Not described
Snider et al. [7]	59	M	MPN with eosinophilia, MPAL	Hydroxyurea, chemotherapy, allo-SCT	Transformation to MPAL 12 months after diagnosis. No evidence of disease 30 days after allo-SCT.
Elnaggar et al. [8]	84	M	CML-like MPD	Imatinib	Lost to follow-up
Schwaab et al. [10]	n/a	n/a	Myeloid neoplasm	Ruxolitinib 20 mg BID, allo-SCT	Complete hematological and cytogenetic remission on ruxolitinib. Relapsed after 18 months upon which referred for allo-SCT.
Schwaab et al. [11]	69	M	MDS/MPN	Ruxolitinib 20 mg BID, allo-SCT	Complete hematological, cytogenetic, and molecular response. AlloSCT while in remission
He et al. [14]	36	F	MPN with eosinophilia	Dasatinib, allo-SCT	No response of dasatinib. No evidence of disease 18 months after allo-SCT.
Bellesso et al. [15]	54	M	*BCR-ABL* ^*-*^ CML	Imatinib, dasatinib, allo-SCT	No response of imatinib or dasatinib. Died of aGvHD 53 days after allo-SCT
Impera et al. [16]	49	F	MPN-unclassifiable	Imatinib, dasatinib peg-interferon	No initial response with imatinib or dasatinib. Partial hematological response with peg-interferon
Lane et al. [17]	44	M	Atypical CML with leukemia cutis	Not described	Not described
Thakral et al. [18]	31	M	MPN with eosinophilia	Hydroxyurea, allo-SCT	No evidence of disease three months posttransplantation

CML, chronic myeloid leukemia; MPD, myeloproliferative disorder; MDS, myelodysplastic syndrome; B-ALL, B-cell acute lymphoblastic leukemia; AML, acute myeloid leukemia; MPN, myeloproliferative neoplasm; MPAL, mixed phenotype acute leukemia; allo-SCT, allogeneic stem cell transplantation; BID, twice a day; aGvHD, acute graft-versus-host disease; F, female; M, male.

## Data Availability

The data used to support the findings of this study are available from the corresponding author upon request.
